# Transcriptional Responses to Pre-flowering Leaf Defoliation in Grapevine Berry from Different Growing Sites, Years, and Genotypes

**DOI:** 10.3389/fpls.2017.00630

**Published:** 2017-05-02

**Authors:** Sara Zenoni, Silvia Dal Santo, Giovanni B. Tornielli, Erica D’Incà, Ilaria Filippetti, Chiara Pastore, Gianluca Allegro, Oriana Silvestroni, Vania Lanari, Antonino Pisciotta, Rosario Di Lorenzo, Alberto Palliotti, Sergio Tombesi, Matteo Gatti, Stefano Poni

**Affiliations:** ^1^Department of Biotechnology, University of VeronaVerona, Italy; ^2^Department of Agricultural Science, University of BolognaBologna, Italy; ^3^Dipartimento di Scienze Agrarie, Alimentari e Ambientali, Università Politecnica delle MarcheAncona, Italy; ^4^Department of Agricultural and Forest sciences, University of PalermoPalermo, Italy; ^5^Dipartimento di Scienze Agrarie, Alimentari e Ambientali, Università di PerugiaPerugia, Italy; ^6^Dipartimento di Scienze delle Produzioni Vegetali Sostenibili, Università Cattolica del Sacro CuorePiacenza, Italy

**Keywords:** grapevine, pre-flowering defoliation, berry transcriptome, flavonoid, secondary metabolite

## Abstract

Leaf removal is a grapevine canopy management technique widely used to modify the source–sink balance and/or microclimate around berry clusters to optimize fruit composition. In general, the removal of basal leaves before flowering reduces fruit set, hence achieving looser clusters, and improves grape composition since yield is generally curtailed more than proportionally to leaf area itself. Albeit responses to this practice seem quite consistent, overall vine performance is affected by genotype, environmental conditions, and severity of treatment. The physiological responses of grape varieties to defoliation practices have been widely investigated, and just recently a whole genome transcriptomic approach was exploited showing an extensive transcriptome rearrangement in berries defoliated before flowering. Nevertheless, the extent to which these transcriptomic reactions could be manifested by different genotypes and growing environments is entirely unexplored. To highlight general responses to defoliation vs. different locations, we analyzed the transcriptome of cv. Sangiovese berries sampled at four development stages from pre-flowering defoliated vines in two different geographical areas of Italy. We obtained and validated five markers of the early defoliation treatment in Sangiovese, an ATP-binding cassette transporter, an auxin response factor, a cinnamyl alcohol dehydrogenase, a flavonoid 3-*O*-glucosyltransferase and an indole-3-acetate beta-glucosyltransferase. Candidate molecular markers were also obtained in another three grapevine genotypes (Nero d’Avola, Ortrugo, and Ciliegiolo), subjected to the same level of selective pre-flowering defoliation (PFD) over two consecutive years in their different areas of cultivation. The flavonol synthase was identified as a marker in the pre-veraison phase, the jasmonate methyltransferase during the transition phase and the abscisic acid receptor PYL4 in the ripening phase. The characterization of transcriptome changes in Sangiovese berry after PFD highlights, on one hand, the stronger effect of environment than treatment on the whole berry transcriptome rearrangement during development and, on the other, expands existing knowledge of the main molecular and biochemical modifications occurring in defoliated vines. Moreover, the identification of candidate genes associated with PFD in different genotypes and environments provides new insights into the applicability and repeatability of this crop practice, as well as its possible agricultural and qualitative outcomes across genetic and environmental variability.

## Introduction

Viticulture is still strongly bound to the concept of *terroir*, relating the sensory attributes of wine to the environmental conditions in which the grapes are grown (Van Leeuwen and Seguin, 2006). Though a shared definition of *terroir* is still hard to find, there is general consensus that the main factors composing *terroir* are climate, soil, cultivar/rootstock and human practices and that these factors strongly interact (Renouf et al., 2010). Quantifying the relative importance of each factor influencing *terroir* is an extremely difficult task since the variability of all factors involved must be represented. A quite considerable effort was made by van Leeuwen et al. (2004) who concluded that climate, soil, and cultivar had a decreasing importance in influencing performance of the cultivars Merlot, Cabernet franc, and Cabernet Sauvignon grown in three different soil environments and observed over 5 years.

A first important consequence of the complex climate × soil × cultivar interactions is that the same cultivar grown in different environments can originate products of different composition and market value. The capacity of a genotype to modulate its phenotype under different environmental conditions is defined phenotypic plasticity, a phenomenon of considerable interest in plant physiology. Over the last decade, a number of studies exploring metabolomic and transcriptomic bases of phenotypic plasticity in *Vitis vinifera* have been conducted in local cultivars such as Corvina and Garganega (Dal Santo et al., 2013a, 2016b; Fernie and Tohge, 2013; Anesi et al., 2015). These works demonstrated the direct effect of growing conditions on gene expression during berry ripening, allowing several environmentally modulated genes to be identified, including many belonging to the phenylpropanoid/flavonoid pathway (Dal Santo et al., 2013a, 2016b). The existence of a *terroir*-specific effect on berry transcriptome and metabolome was also revealed, which persists over several vintages (Anesi et al., 2015), and specific plastic transcripts were associated with groups of vineyards sharing common viticulture practices (Dal Santo et al., 2013a).

If it is agreed that human practices are an important component of the *terroir* concept (Renouf et al., 2010), forecasting their effects on grape composition and wine can greatly benefit from associating omics tools to traditional agronomic assessment. Among the different operations pertaining to grapevine canopy management, pre-flowering leaf removal is likely the one that has received the greatest interest from the scientific community over the last decade. Starting with the original work (Poni et al., 2006), a number of subsequent studies, representing a broad array of cultivars and environments (Diago et al., 2012; Gatti et al., 2012, 2015; Palliotti et al., 2012; Lee and Skinkis, 2013; Pastore et al., 2013; Risco et al., 2014; Komm and Moyer, 2015; Sternad Lemut et al., 2015; Sivilotti et al., 2016), have confirmed the technique to be valuable and repeatable for: (i) reducing vine yield through a decrease in fruit-set and/or berry size; (ii) decreasing cluster compactness, hence susceptibility to rot diseases, and (iii) improving grape composition in terms of total soluble solids (TSSs), phenolic and aroma compounds. However, it was also observed that the outcome of pre-flowering defoliation (PFD) could be quite variable between consecutive years (Gatti et al., 2015) and that different cultivars or the same cultivar in different environments could be influenced differently by the treatment (Kuhn et al., 2014).

Although the practice is widely used in viticulture, very little molecular information is available and, as a consequence, the definition of common mechanisms linking the impact of leaf removal to berry physiological and metabolic responses, is far from complete.

The pioneer study conducted on genome-wide expression analysis in cv. Sangiovese vines subjected to either pre-flowering or late season (i.e., at veraison) defoliation revealed a general delay in transcriptional ripening following both treatments (Pastore et al., 2013). Moreover, a more extensive transcriptome rearrangement in berries subjected to PFD was observed, which reflects the uncoupling of metabolic processes, in particular anthocyanin and flavonol synthesis, from the general ripening program (Pastore et al., 2013). A very recent study performed on Sauvignon blanc shed more light on grapevine response to an altered microclimate due to early leaf removal (Young et al., 2016). When main and lateral leaves were removed from the cluster zone at fruit-set in order to induce and maintain berry light exposure, higher levels of carotenoids and volatile terpenoids were found in the berries, in two consecutive years. The study also clearly demonstrated that the main physiological responses occur in the early stages of berry development, when berries are still photosynthetically active (Palliotti and Cartechini, 2001), and that the key response is the change in pigment levels and metabolite pools that have photoprotective and/or antioxidant functions (Young et al., 2016). Overall, it is clear that early defoliation combined with environmental conditions affects berry composition through changes in gene expression.

The complexity involved in the reprogramming of berry transcriptome, proteome, and metabolome during development has been progressively described in different grapevine varieties (Deluc et al., 2007; Zamboni et al., 2010; Fasoli et al., 2012; Agudelo-Romero et al., 2013; Dal Santo et al., 2013a; Anesi et al., 2015), demonstrating that a large part of metabolic changes characterizing berry formation and ripening are under transcriptional control. It is also known that grape berry development involves the integration of multiple hormonal signals, with some hormones acting as promoters and others as repressors. In particular, in non-climacteric fruits, such as grape, where no burst in ethylene production is observed during ripening, the abscisic acid (ABA) seems to play a stronger role during ripening and its crosstalk with other growth regulators has been proposed at different berry stages (Davies and Bottcher, 2009; McAtee et al., 2013; Fortes et al., 2015). Despite the amount of information already reported, hormonal control in grape ripening is still poorly understood (Fortes et al., 2015). In this context, the identification of molecular markers, addressing the question of how stable and replicable is the link of PFD to favorable physiological and metabolic changes in berry, represents a huge challenge.

In this work, a comparative study of the agronomic and molecular berry responses to PFD was performed in four genotypes grown in different areas of cultivation over two consecutive years, with the aim of identifying genes whose expression could be attributable to this viticulture practice, regardless of site, year, and genotype. Molecular responses in Sangiovese berries during development from defoliated and untreated control vines in two different growing sites were investigated by a genome wide expression analysis. The expression profiles of selected candidate genes were assessed by qPCR in all experimental conditions and integrated with agronomic and ripening parameters, to unveil developmental and metabolic processes commonly affected in berries after PFD.

## Materials and Methods

### Plant Material, Experimental Layout, and Berry Sampling for Gene Expression Analyses

Berry samples for subsequent transcriptomic analyses and real time qPCR analyses were taken from mature and healthy vineyards located in Emilia Romagna (cvs Sangiovese and Ortrugo), Umbria (cv. Ciliegiolo), Marche (cv. Sangiovese), and Sicily (cv. Nero d’Avola), Italy. Sangiovese plots in Emilia Romagna and Marche shared the same rootstock (S.O.4.), whereas clones were different: clone R24 and clone SG12T, respectively. All vineyards were standard either cane or spur pruned vertically shoot positioned (VSP) trellises. Single vine spacing within row varied between 0.8 and 1.5 m, whereas between-row spacing was between 2.5 and 3.3 m, resulting in a vine density varying from 2020 to 5000 vines/hectare. More details regarding trellis structure, bud load, soil characteristics, climate trends and canopy and vineyard management practices can be found in Filippetti et al. (2011), Alagna et al. (2014), Gatti et al. (2015), and Silvestroni et al. (2016).

In 2012 and 2013, in each site × cultivar combination, two treatments were compared consisting of PFD performed at the “separated closed flowers” stage (Baggiolini, 1952) by removing the six basal main leaves of all shoots on each test vine (varying from 6 to 12 according to site; Supplementary Figure 1A) while any lateral shoot emerging from the same basal nodes at the time of defoliation was retained. PFD was compared with a non-defoliated control treatment (C).

Using an identical sampling protocol, berry sampling at each site × cultivar combination was performed on both control and PFD treatments at four development stages as follows: 20 days after leaf removal (Stage 1); hard and green berries at veraison (i.e., 1–5% slightly colored berries in a cluster) (Baggiolini, 1952) (Stage 2); soft, yet still not colored berries at veraison (Stage 3), berries at a TSS concentration of about 18°Brix (Stage 4). On each sampling date, a batch of 60 berries was collected. In detail, three independent pools of 20 berries each were collected from clusters of different vines in order to create three biological replicates that represent almost the entire variability of the experimental design. The sampling was performed by carefully cutting each berry at the pedicel with scissors in order to avoid any damage or juice loss. Berries were immediately frozen in liquid nitrogen and then shipped to labs at the University of Verona (northern Italy) for transcriptome processing and expression analyses. Given the typical asynchrony in individual berry ripening, berry sampling for Stages 2 and 3 were performed on the same date for both treatments.

In total for the transcriptomic analysis on Sangiovese, the experiment entailed the collection and analysis of 48 berry samples (4 stages × 2 treatments × 2 sites × 3 biological replicates). For the real time qPCR on all site × cultivar combinations, the experiment entailed the collection and analysis of 240 berry samples (4 stages × 2 treatments × 5 sites × 3 biological replicates × 2 years).

### Vegetative Growth and Yield Components

For every site × cultivar combination vegetative growth capacity was expressed by estimated total final leaf area per vine and measured pruning weight. Total leaf area per vine was estimated by node counts and surface area of fully expanded main and lateral leaves (Lopes and Pinto, 2015), whereas 1-year pruning weight per vine was taken soon after leaf shedding in fall was completed. At harvest, each year, total yield and cluster number per vine were recorded, and mean cluster weight calculated accordingly. Single berry weight was taken on the samples then processed for must analyses and total berry number calculated from mean cluster weight. For more details on sample size and sampling procedures, please refer to the papers cited above. Source-to-sink balance was expressed as leaf area-to-yield ratio.

### Must Composition and Phenolic Compounds Analyses

Four-to-six 100-berry samples were taken pre-harvest from each genotype by treatment combination in the different test sites. The 100-berry sample was composed by five berries taken for a total of 20 clusters; two berries were sampled from the top portion or wings, two from the middle and one from the tip of the cluster in order to account for within cluster variability in ripening.

TSS concentration, pH, and titratable acidity (TA) were determined on must samples according to standard methods described in Iland et al. (2011).

Total anthocyanin concentration (mg/kg of fresh berry mass) was determined according to Iland (1988).

Flavonol compounds were extracted from grape skins as reported by Downey and Rochfort (2008). In brief: 0.100 g of lyophilized grape skins were extracted in 1.0 mL of 50% (v/v) methanol in water for 20 min with sonication. The extracts were centrifuged (5 min at 10000 ×*g* at 4°C), filtered through a 0.22 μm polypropylene syringe for HPLC analysis and transferred to HPLC auto-sampler vials.

The chromatographic method was developed using an Agilent 1260 Infinity Quaternary LC (Agilent Technology, Santa Clara, CA, USA) consisting of a G1311B/C quaternary pump with inline degassing unit, G1329B autosampler, G1330B thermostat, G1316B thermostatted column compartment and a G4212B diode array detector fitted with a 10 mm path, 1 μL volume Max-Light cartridge flow cell. The instrument was controlled using Agilent Chemstation software version A.01.05. Separation was achieved on a reverse-phase C-18 Synergi Hydro RP 80A, 250 mm × 4.6 mm, 4 μm (Phenomenex, Torrance, CA, USA). The solvents used were 5% (v/v) formic acid (solvent A) and acetonitrile (solvent B). The flow rate was 0.5 mL/min, with a linear gradient profile consisting of solvent A with the following proportions (v/v) of solvent B: 0–10 min, 2–10% B; 10–25 min, 10–12% B; 25–35 min, 12–30% B; 35–43 min, 30% B; 43–48 min, 30–40% B; 48–52 min, 40–50% B; 52–55 min, 50–60% B; 55–58 min, 60–98% B; 58–63 min, 98% B; 63–66 min, 98–2% B; 66–72 min 98% B. The column temperature was maintained at 40 ± 0.1°C. Five microliters of sample extract was injected. The elution was monitored at 200–700 nm, detection by UV-Vis absorption with DAD scanning between 280, 320, and 370 nm. Anthocyanins and flavonols were identified using authentic standards and by comparing the retention times. Quantification was based on peak areas and performed by external calibration with standards.

### Statistical Analyses

A completely randomized block design was used and the agronomic parameters and must composition were subjected to analysis of variance (ANOVA, SAS statistical software, SAS Institute, Cary, NC, USA) and mean separation performed by *t*-test. In other cases, variability across treatments was expressed as mean ± standard error (SE).

### RNA Extraction

Total RNA was isolated from approximately 400 mg of berry pericarp tissue (i.e., entire berries without seeds) using the Spectrum^TM^ Plant Total RNA kit (Sigma–Aldrich), with modifications as described in Dal Santo et al. (2016a). Seeds were manually removed from the 20 berries of each biological replicate before the liquid nitrogen grinding procedure. RNA quality and quantity were determined using a Nanodrop 2000 spectrophotometer (Thermo Scientific, Wilmington, DE, USA) and a Bioanalyzer Chip RNA 7500 series II (Agilent, Santa Clara, CA, USA).

### Microarray Analyses and Statistical Approaches

We hybridized 5 μg of total RNA per sample to a NimbleGen microarray 090818_Vitus_exp_HX12 chip (Roche, NimbleGen Inc., Madison, WI, USA), representing 29,549 predicted genes on the basis of the 12X grapevine V1 gene prediction version. The hybridization was performed according to the manufacturer’s instructions (Dal Santo et al., 2016a). Statistical analysis of the microarray data was conducted using TMeV v4.8 (mev.tm4.org/). Statistical analysis of microarrays (SAM) was conducted with a false discovery rate (FDR) of 0.1% and ANOVA using α = 0.01 and standard Bonferroni correction in order to skim off genes that showed a high variability among the three biological replicates. Correlation matrixes were prepared using R software and Pearson’s correlation coefficient as statistical metric to compare the values of the whole transcriptome (29,549 genes) in all analyzed samples. Correlation values were converted into distance coefficients to define the height scale of the dendrogram. Principal component analysis (PCA) was conducted using SIMCA P+ v13 (Umetrics, USA) and applied to the significantly modulated transcripts dataset (18,771 genes). Differentially modulated genes at each developmental stage in both sites were retrieved by performing a between-subjects (C vs. PFD samples) *t*-test (α = 0.01), assuming equal variance among samples. Gene Ontology (GO) enrichment analysis was performed with the AgriGO online software^1^, using Singular Enrichment Analysis (SEA) tool and Fisher’s as statistical test method (Du et al., 2010). Heat maps were created using log2-transformed expression values and then median-centered by transcript. Cluster analysis was conducted on transcript median-centered fluorescent values by the k-means method (KMC) with Pearson’s correlation distance. We used the Figure of Merit (FOM) statistic to determine the optimal number of clusters (*n* = 10).

### Reverse Transcription (RT) and Real-Time qPCR

One microgram of total RNA was treated with DNase I (Promega) according to the instructions provided with the commercial kit. DNase treated RNA was then used for cDNA synthesis using the Improm-II TMReverse Transcriptase (Promega) following the producer’s indications. The transcriptional profile was analyzed by real-time RT-PCR as described by Zenoni et al. (2011), using the SYBR Green PCR master mix (Applied Biosystems) and a Mx3000P real-time PCR system (Stratagene). Each expression value, relative to VvUBIQUITIN1 (VIT_16s0098g01190), widely used as a suitable and robust reference gene during berry development (Chen et al., 2013; Dal Santo et al., 2013b, 2016b; Cramer et al., 2014), was determined in triplicate. Non-specific PCR products were identified by the dissociation curves. Amplification efficiency was calculated from raw data using LingRegPCR software (Ramakers et al., 2003). The mean normalized expression (MNE)-value was calculated for each sample referred to the ubiquitin expression according to the Simon equation (Simon, 2003). Standard error (SE)-values were calculated according to Pfaffl et al. (2002). The primer sequences used in qPCR analysis are listed in Supplementary Table 1.

### Analysis of Correlation between Microarray and qPCR

Correlation between the microarray and qPCR results was performed for the six putative molecular markers of the PFD treatment in Sangiovese for the year 2012, and the statistical significance of this correlation determined. For the NimbleGen microarray, the data input into the correlation analysis was the Log2 value of the average of the three biological replicates for each gene × site × treatment combination. For qPCR, we used the mean Log2 ratio value reported by qPCR from all replicate. Prior to performing correlation analyses, the data were tested for normality using the Shapiro–Wilk test, as indicated by Morey et al. (2006). Because the data were not normally distributed, Spearman’s Rho, instead of Pearson’s correlation, was computed using R software. The calculated correlation coefficient was 0.4617597 (Spearman’s Rho, *p* = 2.185e-06, *n* = 96). By normal standards [*n* = (96 – 2) = 94], the correlation between the NimbleGen microarray data and qPCR data for the indicated six genes, would be considered statistically significant (*p* < 0.005).

### Accession Numbers

Grape berry microarray expression data are available in the Gene Expression Omnibus under the series entry GSE92980^2^.

## Results

### Impact of Pre-flowering Defoliation on Agronomic Parameters and Berry Transcriptome of cv. Sangiovese under Two Growing Conditions

In order to provide a preliminary evaluation of site × early defoliation interaction, the cv. Sangiovese was subjected in 2012 to the pre-flowering defoliation treatment (PFD) using the same protocol in Ancona (AN) and Bologna (BO) and, within each site, a non-defoliated control treatment (C) was also included.

Daily maximum, minimum, and mean air temperatures (T) as well as daily rainfall for the two locations evaluated from the 1 April until 30 September, showed some common features for the two sites, with a quite cool spring and long summer (Supplementary Figure 1B).

Agronomic parameters showed that the two sites shared significant differences for total leaf area and yield per vine, cluster weight, and berries per cluster between PFD and C treatments, with PFD showing lower values than C vines in all cases (**Table 1**). The remaining parameters, including the source–sink balance expressed as leaf area-to-yield ratio were either unchanged or slightly enhanced in AN, as concerns TA and total anthocyanins concentration.

**Table 1:** Agronomic and ripening parameters 2012.

	Bologna			Ancona		
	Control	PFD	F-prob	Control	PFD	F-prob
**Agronomic parameters**						
LA (m^2^)	6.0	4.1	^∗∗^	3.56	2.81	^∗^
Pruning weight (Kg)	0.51	0.58	ns	0.52	0.51	ns
Yield (Kg)	6.46	5.41	^∗^	2.14	1.37	^∗^
LA/yield (m^2^/Kg)	0.98	0.78	ns	1.98	1.90	ns
Cluster weight (g)	332	288	^∗^	181	130	^∗∗^
Berry weight (g)	2.30	2.10	ns	1.78	1.63	ns
Berries/cluster	144	137	^∗^	102	80	^∗^
**Ripening parameters**						
TSS (Brix)	22.1	22.7	ns	24.7	25.0	ns
pH	3.40	3.43	ns	3.45	3.41	ns
TA (g/L)	7.0	7.01	ns	6.14	6.85	^∗^
Anthocyanin (mg/kg)	667	704	ns	844	972	^∗^

*Vegetative growth, yield parameters, and grape composition recorded in 2012 on cv. Sangiovese grapevines grown in Ancona and Bologna and subjected, within each location, to a pre-flowering defoliation (PFD) or undefoliated (Control). ^∗^P < 0.05 and ^∗∗^P < 0.01, respectively.*

**FIGURE 1:**
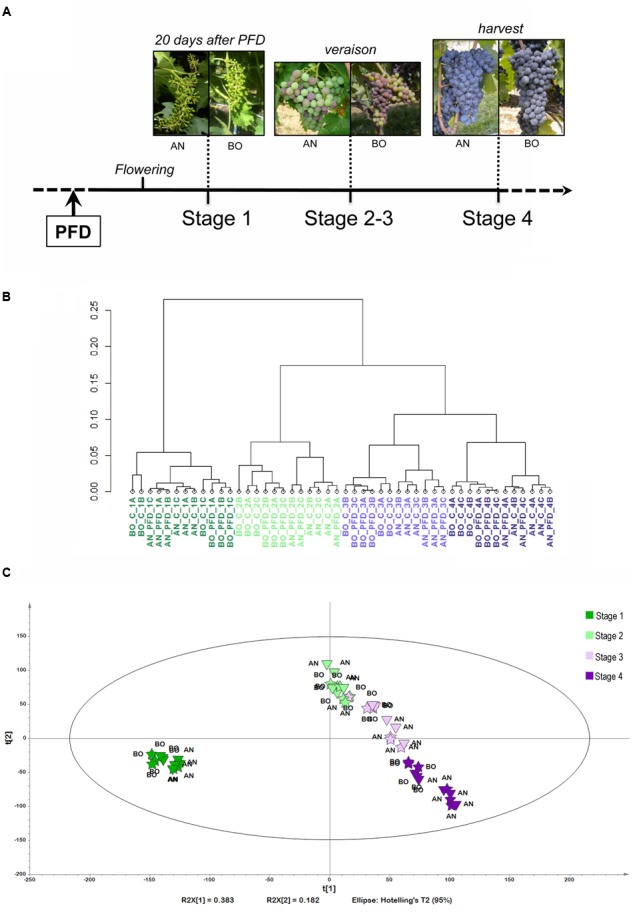
**Whole transcriptome analysis of Sangiovese berries subjected to PFD treatment in two different sites. (A)** Schematic representation of the sampling design used. **(B)** Cluster dendrogram of the whole transcriptome dataset in all analyzed samples. Pearson’s correlation values were converted into distance coefficients to define the height of the dendrogram. Samples are colored according to the developmental stage of sampling. **(C)** Score scatterplot (PC1 vs. PC2) of the PCA model (9 Principal Components, *R*^2^(cumulative) = 0.903, *Q*^2^(cumulative) = 0.848) applied to the significantly modulated transcripts dataset. Samples are colored according to the developmental stage of sampling. Different treatments are indicated by different symbols, “ding73” = Control and “
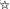
” = Pre-flowering defoliation.

To investigate the molecular changes that take place after defoliation during berry development, Sangiovese berry transcriptomes of PFD and C vines were compared at the four berry developmental stages in both sites (**Figure 1A**). A dendrogram of the global transcriptomic data revealed enough uniformity among the three biological replicates in the C and PFD samples at each time point (**Figure 1B**). The main separation among samples is related to berry stage, with Stage 1 resulting as the most divergent. Indeed, Stage 2 showed more similarity at the transcriptional level to Stages 3 and 4, suggesting that, despite berries still being hard and green at Stage 2, many molecular processes related to ripening were already activated. Interestingly, the second variable that strongly influenced sample association was site. In fact, except for Stage 1, AN and BO samples were characterized by distinctive berry transcriptomes during ripening. This evidence highlights the strong effect of growing conditions on berry transcriptome in Sangiovese and suggests that this effect is more evident when the ripening program is initiated.

Concerning the effect of the defoliation treatment on berry transcriptome we found that PFD and C vines were distinguishable only at specific combinations of berry stage and site. In fact, the separation between PFD and C is evident at Stage 1 in AN, at Stage 2 in BO, at Stage 3 in AN and at Stage 4 in BO. These results suggest that, in Sangiovese, PFD has a weaker effect on berry transcriptome than growing conditions.

To retrieve genes differentially modulated under our experimental conditions, the berry transcriptome dataset was screened by significance analysis of microarrays (SAM, 16 groups, FDR = 0.1%). Analysis of variance (ANOVA, 16 groups, α = 0.01, standard Bonferroni correction) was applied to transcripts positive in the previous SAM experiment in order to skim off the most significantly modulated transcripts. We obtained a reduced dataset of 18,771 genes (Supplementary File 1), which was inspected by PCA analysis. The two principal components, explaining 56.5% of the total dataset variability, allowed berry samples to be clearly separated on the basis of their developmental stage (**Figure 1C**). Sample distribution confirmed that Stage 2 is more similar to Stage 3 at both sites; moreover, it can be observed that, at transcriptional level, the ripening process (Stages 3 and 4) at AN is slightly advanced in comparison to BO (**Figure 1C**). Interestingly, principal component 3 (PC3), explaining 11.8% of the total dataset variability, clearly separated AN from BO samples, in particular after veraison, again evidencing the strong effect of growing conditions on the berry transcriptome rearrangement during ripening in cv. Sangiovese (Supplementary Figure 2). At no stage nor in either site were principal components found that separated PFD samples from C ones.

**FIGURE 2:**
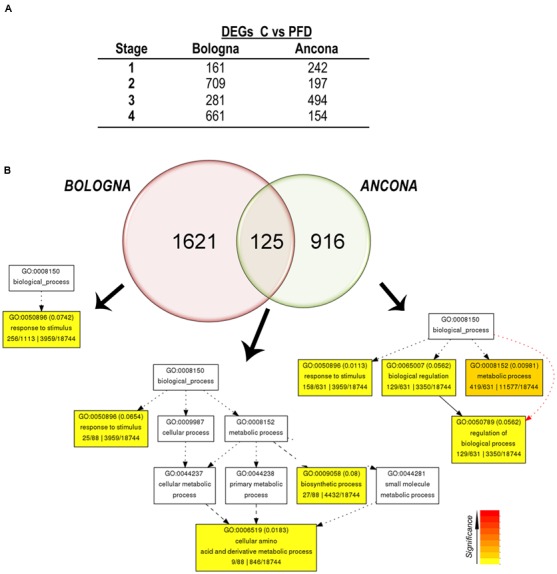
**Transcriptomic responses to PFD treatment in two different sites. (A)** Differentially expressed genes (*t*-test with a α = 0.01) between C and PFD vines at each sampling time point in the two sites. **(B)** Venn diagram summarizing results obtained in **(A)** was constructed using Venny 2.1.0 and redrawn. **(C)** Gene Ontology (GO) enrichment analysis was performed, using the AgriGO online software (Du et al., 2010), on Bologna-, Ancona-, and common PFD modulated transcripts separately. Statistically significant GO categories are highlighted in color, according to the given significance color-key.

Notwithstanding the small effect of PFD on berry transcriptome, we focused on the identification of differentially expressed genes (DEGs) after PFD, regardless of the growing site. We then compared the PFD and C berry transcriptomes at each time point using a *t*-test (between subjects *t*-test, α = 0.01) for both sites separately. The number of DEGs identified between PFD and C vines at each stage was different in the two sites (**Figure 2A** and Supplementary File 2). In particular, at Stage 1 and 3 a higher number of DEGs characterized AN, whereas at Stage 2 and 4 a higher number of DEGs was found in BO. These differences well mirrored the behavior observed in the dendrogram analysis. A total of 1746 and 1041 DEGs in at least one stage between PFD and C vines, were found in BO and AN, respectively.

By comparing the list of DEGs from BO and AN, only 125 genes were identified as differentially expressed at both sites (**Figure 2B** and Supplementary File 3). The GO enrichment analysis performed on the three group of DEGs, i.e., BO-specific, AN-specific and common, revealed a significant overrepresentation of the “response to stimulus” functional category in all groups of DEGs. Genes belonging to this functional category may be involved in the detection and response to external and endogenous stimuli and also to many stresses, such as biotic and abiotic stress, redox state, and others. Regarding the common DEGs, another two functional categories resulted as significantly overrepresented, the “biosynthetic process,” and “cellular amino acid and derivative metabolic process,” represented by an aspartate aminotransferase, two glycine hydroxymethyltransferases, a glutamine synthetase, and a serine hydroxymethyltransferase.

### Identification of Berry Molecular Markers Associated to Pre-flowering Defoliation in Sangiovese

**FIGURE 3:**
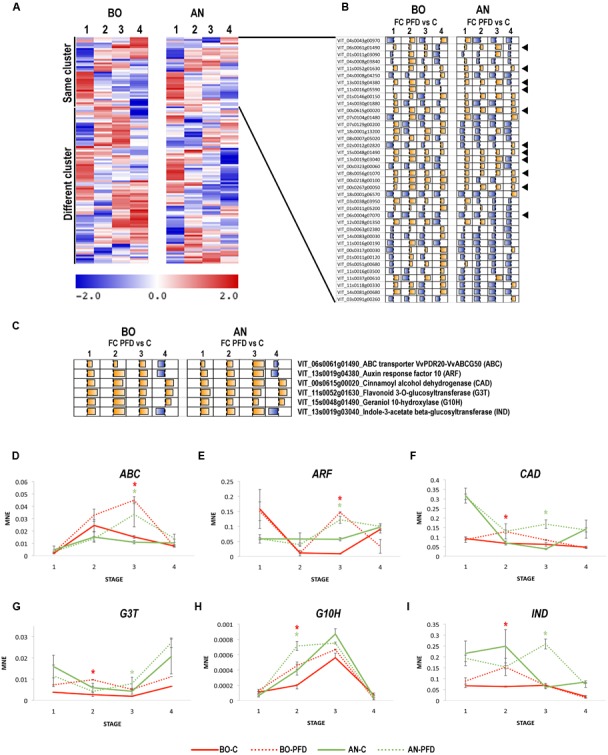
**PFD treatment molecular markers of Sangiovese cultivar selection and real-time qPCR validation in 2012. (A)** Heat map representing the fluorescence intensity of C vines in the 125 commonly modulated genes. KMC analysis was used to determine the transcripts with unaltered expression between Bologna and Ancona sites (highlighted as *same cluster*) and those with different expression (highlighted as *different cluster*). **(B)** Schematic representation of the Fold Change (FC), calculated between C and PFD vines at each developmental stage in Ancona and Bologna, in the 38 *same cluster* transcripts found in **(A)**. The black arrows indicate the 11 genes showing a similar trend of FC. **(C)** FC between C and PFD vines at each developmental stage in Ancona and Bologna in a selection of six transcripts. **(D–I)** Real-time qPCR validation of the **(D)** ABC transporter VvPDR20-VvABCG50 (*ABC*; VIT_06s0061g01490), **(E)** Auxin response factor 10 (*ARF*; VIT_13s0019g04380), **(F)** Cinnamoyl alcohol dehydrogenase (*CAD*; VIT_00s0615g00020), **(G)** Flavonoid 3-*O*-glucosyltransferase (*G3T*; VIT_11s0052g01630), **(H)** Geraniol 10-hydroxylase (*G10H*; VIT_15s0048g01490) and **(I)** Indole-3-acetate beta-glucosyltransferase (*IND*; VIT_13s0019g03040) expression profiles in PDF and C Sangiovese vines during berry development in 2012. The mean normalized expression (MNE)-value was calculated for each sample referred to the VvUBIQUITIN1 (VIT_16s0098g01190) expression according to the Simon equation (Simon, 2003). Bars represent means ± SE of three biological replicates. The significant modulation (*t*-test, *p* < 0.05) of gene expression between C and PFD berries at each stage per each site is indicated by an asterisk, red for BO and green for AN.

In order to identify putative molecular markers associated to the PFD in cv. Sangiovese, independently of site, we focused on the 125 DEGs shared by BO and AN. We checked the expression profile of the 125 genes during berry development on C vines in the two sites, in order to identify genes whose expression was not or slightly influenced by the growing conditions. A KMC clustering analysis identified 10 expression clusters and revealed a very high expression variability of these genes in C vines growing in the two sites, with only 38 genes belonging to same clusters of expression during development in BO and AN (**Figure 3A** and Supplementary File 4). Subsequently, we evaluated the pattern of Fold change (FC) between PFD and C at each stage in both sites, and we found that only 11 genes were affected by the PFD in a similar manner throughout berry development in BO and AN (**Figure 3B**). Among these genes we selected six characterized by an upregulation at all stages after the PFD treatment or by an upregulation till Stage 3 and a downregulation at Stage 4 (**Figure 3C**). The selected genes were represented by the ATP-binding cassette (ABC) transporter VvPDR20/VvABCG50 (VIT_06s0061g01490), auxin response factor (ARF) 10 (VIT_13s0019g04380), cinnamyl alcohol dehydrogenase (CAD; VIT_00s0615g00020), flavonoid 3-*O*-glucosyltransferase (G3T; VIT_11s0052g01630), geraniol 10-hydroxylase (G10H; VIT_02s0012g02820), and indole-3-acetate beta-glucosyltransferase (IND; VIT_13s0019g03040). The expression of these genes were validated by qPCR on PFD and C berries in the two sites at the four berry developmental stages (**Figures 3D–I**). The VvPDR20/VvABCG50 showed a peak of expression at Stage 2 in C berries throughout development, whereas in PFD berries its expression significantly increased at Stage 3 in both sites (**Figure 3D**). Although very few functional studies have been performed on ABC transporters activity in grape, a role in the vacuolar localization and transport of glucosylated anthocyanidins was demonstrated for the member VvABCC1 (Francisco et al., 2013). The expression profiles obtained by our analysis suggest a role of VvPDR20/VvABCG50 at the onset of ripening and evidenced that PFD delays its expression during berry development. In the case of the ARF the increase in expression at Stage 4 in C condition is significantly hastened by the PFD treatment at Stage 3 in both sites (**Figure 3E**). The CAD gene is characterized by a significant increase of expression at Stage 3 in PFD condition instead of the Stage 4 observed in C vines in AN, and by a significant increase at Stage 2 in PFD in both sites, particularly in BO (**Figure 3F**). This behavior well mirrored the trend observed in the dendrogram, showing that the separation between PFD and C is more evident at Stage 2 in BO, and at Stage 3 in AN. The G3T was characterized by an increase in expression at Stage 4 in C condition in both growing sites. For this gene the PFD treatment led to a significant increase of expression level throughout berry development, in particular at Stage 2 in BO and Stage 3 in AN, similarly to the CAD (**Figure 3G**). The G10H showed a peak of expression at Stage 3 in C vines in both growing sites. The PFD treatment enhances the expression level of this gene at Stage 3 but also significantly hastens its induction at Stage 2 in both BO and AN (**Figure 3H**). Concerning the IND, involved in the regulation of auxin levels by IAA conjugation (Bottcher et al., 2010; Fortes et al., 2015), a flat expression trend during the first stages of berry development with a slight downregulation at Stage 4 was found in BO C vines, whereas a high expression was observed at Stages 1 and 2 in AN, followed by a decrease in expression after veraison. The PFD induced a significant increase of IND expression in both sites but at Stage 2 in BO and Stage 3 in AN (**Figure 3I**).

Overall real-time qPCR analysis confirmed the microarray expression profiles for all the selected genes, demonstrating that these genes could be considered putative molecular markers of PFD in berry throughout development in cv. Sangiovese in 2012.

In order to investigate the influence of year on the PFD effect, pre-flowering leaf removal was also applied at both sites in 2013 using the same protocol. Seasonal weather data as daily air temperature and rainfall are shown in Supplementary Figure 1B.

Leaf area and yield per vine, cluster weight, and berries per cluster were again significantly lower in PFD vines, whereas pruning weight per vine and leaf area-to-yield ratio were not modified (**Table 2**). A striking berry size reduction was recorded in BO, while TSS were notably higher in PFD in both locations. However, while increased TSS did not achieve a concurrent increase in anthocyanins at the AN site, total anthocyanins were significantly higher in the defoliated vines at BO (**Table 2**).

**Table 2:** Agronomic and ripening parameters 2013.

	Bologna			Ancona		
	Control	PFD	F-prob	Control	PFD	F-prob
**Agronomic parameters**						
LA (m^2^)	4.07	2.33	^∗∗^	3.73	2.88	^∗^
Pruning weight (Kg)	0.63	0.58	ns	0.63	0.57	ns
Yield (Kg)	6.79	4.27	^∗^	5.6	3.9	^∗^
LA/yield (m^2^/Kg)	0.60	0.54	ns	0.69	0.77	ns
Cluster weight (g)	548	322	^∗∗^	371	279	^∗∗^
Berry weight (g)	2.73	2.12	^∗∗^	2.52	2.70	ns
Berries/cluster	200	152	^∗^	147	103	^∗^
**Ripening parameters**						
TSS (Brix)	21.3	23.0	^∗∗^	21.0	23.1	^∗∗^
pH	3.30	3.34	ns	3.27	3.33	^∗^
TA (g/L)	7.9	7.7	ns	7.85	7.61	ns
Anthocyanin (mg/kg)	574	745	^∗∗^	530	553	ns

*Vegetative growth, yield parameters, and grape composition recorded in 2013 on cv. Sangiovese grapevines grown in Ancona and Bologna and subjected, within each location, to a PFD or undefoliated (Control). ^∗^P < 0.05 and ^∗∗^P < 0.01, respectively.*

**FIGURE 4:**
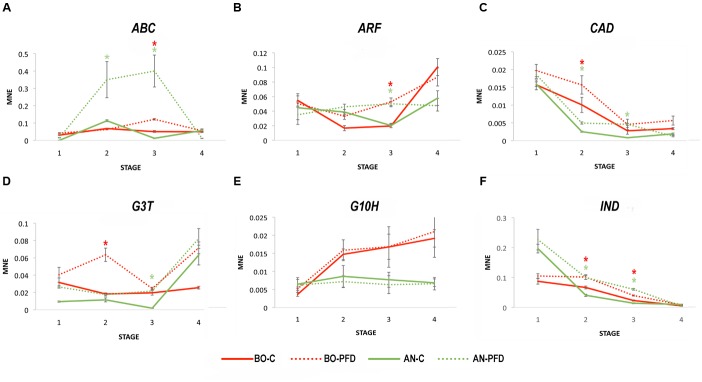
**Real-time qPCR analysis of PFD treatment molecular markers of Sangiovese cultivar in 2013.** Real-time qPCR analysis of the **(A)** ABC transporter VvPDR20-VvABCG50 (*ABC*; VIT_06s0061g01490), **(B)** Auxin response factor 10 (*ARF*; VIT_13s0019g04380), **(C)** Cinnamoyl alcohol dehydrogenase (*CAD*; VIT_00s0615g00020), **(D)** Flavonoid 3-*O*-glucosyltransferase (*G3T*; VIT_11s0052g01630), **(E)** Geraniol 10-hydroxylase (*G10H*; VIT_15s0048g01490) and **(F)** Indole-3-acetate beta-glucosyltransferase (*IND*; VIT_13s0019g03040) expression profiles in PDF and C Sangiovese vines during berry development in 2013. The mean normalized expression (MNE)-value was calculated for each sample referred to the VvUBIQUITIN1 (VIT_16s0098g01190) expression according to the Simon equation (Simon, 2003). Bars represent means ± SE of three biological replicates. The significant modulation (*t*-test, *p* < 0.05) of gene expression between C and PFD berries at each stage per each site is indicated by an asterisk, red for BO and green for AN.

Berry samples were collected following the same protocol used in 2012. The expression of the six genes identified as markers of PFD was analyzed on C and PFD berries collected during 2013 by the qPCR approach. The ABC transporter gene (ABC) showed the same expression profile as that revealed in 2012 in C and PFD vines, with a with a significant induction at Stage 3 in both PFD vines and a very high expression during PFD berry development in AN (**Figure 4A**). The ARF expression was confirmed in 2013 in C and PFD berries in both sites (**Figure 4B**), as well as the effect of PFD on CAD expression (**Figure 4C**). The expression of CAD in C berries was instead slightly different from 2012 in BO, with a clear decrease of expression from Stage 1 throughout berry development not observed the year before. The effect on G3T expression in PFD berries also resulted as the same in the 2 years, again with the significant and stronger effect at Stage 2 in BO and Stage 3 in AN (**Figure 4D**). On the contrary, the expression of GH10 gene was strongly affected by year in C berries and not influenced by PFD treatment in either site (**Figure 4E**). Lastly, beside the different expression in C vines at Stage 2 in AN, IND gene showed the same trend of significant induction as in 2012 due to the PFD treatment in both sites (**Figure 4F**).

Overall, the five genes, ABC, ARF, CAD, G3T, and IND resulted as being putative molecular markers of PFD treatment in Sangiovese during berry development, independently of growing site and year.

### Identification of Putative Molecular Markers of Pre-flowering Defoliation in Different Genotypes

In order to evaluate if the five putative Sangiovese marker genes could also represent molecular markers of PFD for other genotypes cultivated in different environments, we applied PFD using the same protocol adopted for Sangiovese, on Nero d’Avola (ND), Ortrugo (OR), and Ciliegiolo (CI) cultivars, cultivated during 2012 and 2013in three different Italian areas, Palermo (PA-Sicily), Perugia (PE-Umbria) and Piacenza (PI-Emilia Romagna), respectively.

The environmental parameters recorded during 2012 and 2013 at the three sites are reported in the Supplementary Figure 3. In general, for sites located in north and central Italy (PI and PE) yearly weather patterns and rainfall showed some common features while the Sicilian location (PA) had a quite different trend. As observed for BO and AN, 2012 was marked by a quite cool spring and long summer (mid-June till end of August) in PI and PE, with hot spells reaching 40°C and very limited rainfall (Supplementary Figures 1, 3). In PA, both seasons showed a more progressive increase in air temperature peaking around 40°C in 2012 with basically no rainfall. 2013 was slightly cooler with some rain falling at the end of the season.

In 2012, yield per vine was significantly reduced by PFD regardless of cultivar, although responsiveness of some yield components showed variability (i.e., unchanged berry weight in Ciliegiolo and unchanged cluster weight and berries per cluster in Ortrugo) (**Table 3**). TSS at harvest were always increased by PFD in all cultivars and the same response was seen for total anthocyanins in cvs Ciliegiolo and Nero d’Avola. Except for the white cv. Ortrugo, must pH and TA were less responsive overall, whereas final LA/yield ratio was in general slightly enhanced in PFD.

**Table 3:** Agronomic and ripening parameters 2012.

	Nero D’Avola			Ortrugo			Ciliegiolo		
	Control	PFD	F-prob	Control	PFD	F-prob	Control	PFD	F-prob
**Agronomic parameters**									
LA (m^2^)	3.8	4.6	^∗∗^	3.5	3.52	ns	3.9	3.8	ns
Pruning weight (Kg)	0.8	0.84	ns				0.56	0.58	ns
Yield (Kg)	3.33	2.5	^∗^	3.64	2.65	^∗^	3.45	2.78	^∗^
LA/yield (m^2^/Kg)	1.1	1.8	^∗^	0.95	1.84	ns	1.09	1.36	ns
Cluster weight (g)	222	166.5	^∗^	298	256	ns	200	175	^∗∗^
Berry weight (g)	1.46	1.26	^∗^	2.13	1.92	^∗∗^	1.66	1.79	ns
Berries/cluster	141	121	^∗^	140	133	ns	128	101	^∗^
**Ripening parameters**									
TSS (Brix)	20	22	^∗∗^	18.3	20.8	^∗∗^	22.6	23.7	^∗^
pH	3.7	3.7	ns	3.28	3.19	^∗^	3.34	3.4	ns
TA (g/L)	7.0	6.8	ns	5.55	4.88	^∗∗^	6.07	5.95	ns
Anthocyanin (mg/kg)	570.2	499	^∗^				820	1071	^∗∗^

*Vegetative growth, yield parameters, and grape composition recorded in 2012 on cv. Nero d’Avola, Ortrugo, and Ciliegiolo grapevines grown in Sicily, Emilia Romagna, and Umbria, respectively, and subjected, within each location, to a PFD or undefoliated (Control). ^∗^P < 0.05 and ^∗∗^P < 0.01, respectively.*

In 2013, yield per vine and cluster weight were reduced in PFD regardless of location (**Table 4**). In agreement with the 2012 response, TSS and total anthocyanins were higher in the defoliated vines across all cultivars. Must pH and TA confirmed their relatively low sensitivity to the applied treatment, whereas moderately higher LA/yield ratios were again found in the PFD vines.

**Table 4:** Agronomic and ripening parameters 2013.

	Nero d’Avola			Ortrugo			Ciliegiolo		
	Control	PFD	F-prob	Control	PFD	F-prob	Control	PFD	F-prob
**Agronomic parameters**									
LA (m^2^)	3.86	4.23	^∗∗^	5.17	4.27	ns	4.20	4.0	ns
Pruning weight (Kg)	0.76	0.80	ns				0.67	0.63	ns
Yield (Kg)	4.93	3.72	^∗^	4.51	3.39	^∗∗^	3.86	3.04	^∗^
LA/yield (m^2^/Kg)	0.8	1.1	^∗^	0.87	1.03	ns	1.15	1.34	ns
Cluster weight (g)	290	196	^∗^	315	238	^∗∗^	219	188	^∗∗^
Berry weight (g)	1.84	1.25	^∗^	1.94	1.82	^∗^	1.76	1.8	ns
Berries/cluster	148	145	ns	162	131	ns	118	104	ns
**Ripening parameters**									
TSS (Brix)	21.6	20.5	^∗^	20.2	22.6	^∗∗∗^	22.3	24.4	^∗^
pH	3.3	3.4	ns	3.04	3.18	^∗∗∗^	3.34	3.37	ns
TA (g/L)	6.5	6.7	ns	5.94	5.14	^∗∗∗^	6.0	5.75	ns
Anthocyanin (mg/kg)	591.7	756.3	^∗∗^				834	1124	^∗∗^

*Vegetative growth, yield parameters, and grape composition recorded in 2013 on cv. Nero d’Avola, Ortrugo, and Ciliegiolo grapevines grown in Sicily, Emilia Romagna, and Umbria, respectively, and subjected, within each location, to a PFD or undefoliated (Control). ^∗^P < 0.05, ^∗∗^P < 0.01, and ^∗∗∗^P < 0.001, respectively.*

Berry samples for gene expression analysis were collected from C and PFD vines at the same four phenological stages used for Sangiovese. The real-time qPCR of the five Sangiovese PFD molecular markers performed on berries collected during 2012 showed that the five genes were not differentially modulated nor showed different expression profiles during berry development in Nero d’Avola, Ortrugo, and Ciliegiolo subjected to PFD (data not shown). These results demonstrated that these genes could not be considered molecular markers of PFD for other genotypes and/or for other environmental conditions. The transcriptomic dataset of 18771 genes obtained for Sangiovese was therefore inspected again with the aim of finding genes consistently modulated by PFD at the same stage in both sites, without necessarily showing a similar expression profile throughout the entire berry development. By using a threshold of | FC | > 2 between C and PFD we found that 6 genes were consistently modulated by PFD at Stage 1, 28 genes at Stage 2, 39 at Stage 3, and 19 at Stage 4 in both sites (Supplementary File 5). Among genes modulated at Stage 1, 5 resulted as upregulated by PFD and only 1, an unknown protein, downregulated. The flavonol synthase (VIT_18s0001g03470) resulted as the most induced by the treatment in both sites and was therefore selected for further transcriptional investigation in other terroirs, years, and genotypes (Supplementary File 5). At Stage 2, 16 genes resulted as commonly upregulated by PFD, including the already analyzed geraniol 10-hydroxylase (G10H), some genes involved in carbohydrate metabolism, the MADS-box AGL20 (VIT_15s0048g01240) and jasmonate *O*-methyltransferase (VIT_18s0001g12890). Among the 12 genes downregulated by the treatment we found three chitinases, one pathogenesis-related protein and the indol-3-acetic acid amino synthetase (Supplementary File 5). We selected the MADS-box AGL20 and jasmonate *O*-methyltransferase for further analysis. Interestingly, at Stage 3 all the 39 commonly modulated genes resulted as upregulated by PFD. Among these genes at least 10 terpene synthases were found, together with three multidrug resistance-associated proteins, three serine carboxypeptidases and two genes involved in jasmonate metabolism, the VvJAZ2 and jasmonate *O*-methyltransferase, upregulated also at Stage 2 (Supplementary File 4). For further investigation, we chose one multidrug transporter (ABC- VIT_09s0020g05380), the linalool synthase VvTPS62 (VIT_00s0572g00020) and jasmonate *O*-methyltransferase. Finally, the 19 genes commonly modulated by PFD at Stage 4 all resulted as downregulated in comparison to the C vine. Among these genes we found three kinases, two NAC transcription factors and the ABA receptor PYL4 (VIT_08s0058g00470) that were the most downregulated (Supplementary File 5). The latter was chosen for the next investigation.

**FIGURE 5:**
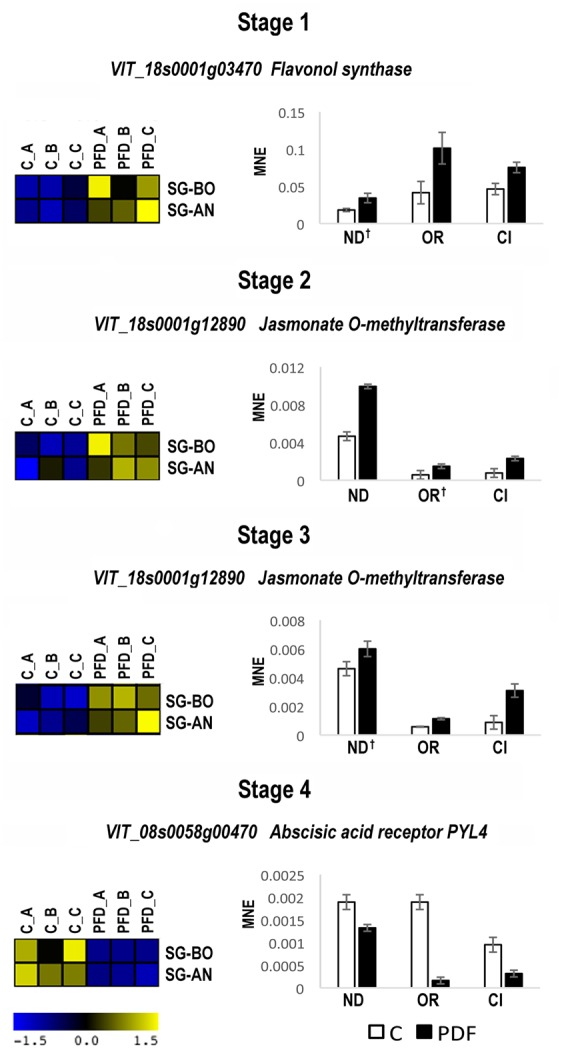
**Real-time qPCR analysis of PFD treatment common molecular markers in 2012.** Real-time qPCR analysis of the flavonol synthase (VIT_18s0001g03470) at Stage 1, jasmonate *O*-methyltransferase (VIT_18s0001g12890) at Stages 2 and 3 and abscisic acid receptor PYL4 (VIT_08s0058g00470) at Stage 4 of berry development from PDF and C vines of Nero d’Avola, (ND), Ortrugo (OR) and Ciliegiolo (CI) in 2012. The mean normalized expression (MNE)-value was calculated for each sample referred to the VvUBIQUITIN1 (VIT_16s0098g01190) expression according to the Simon equation (Simon, 2003). Bars represent means ± SE of three biological replicates. All genes in all genotypes resulted significantly modulated (*t*-test; *p* < 0.05) between C and PFD berries. The † indicates no significance. Heat maps reported on the left of each real-time qPCR represent the fluorescence intensity of the genes in PFD and C Sangiovese vines in Bologna (SG-BO) and Ancona (SG-AN) obtained by microarray analysis in 2012. A, B, and C correspond to the three biological replicates at each stage.

**FIGURE 6:**
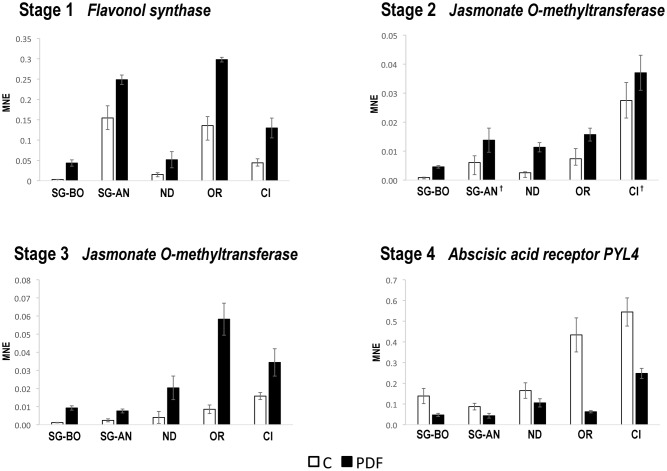
**Real-time qPCR analysis of PFD treatment common molecular markers in 2013.** Real-time qPCR analysis of the flavonol synthase (VIT_18s0001g03470) at Stage 1, jasmonate *O*-methyltransferase (VIT_18s0001g12890) at Stages 2 and 3 and abscisic acid receptor PYL4 (VIT_08s0058g00470) at Stage 4 of berry development from PDF and C vines of Sangiovese at Bologna (SG-BO) and Ancona (SG-AN) sites, Nero d’Avola, (ND), Ortrugo (OR) and Ciliegiolo (CI) in 2013. The mean normalized expression (MNE)-value was calculated for each sample referred to the VvUBIQUITIN1 (VIT_16s0098g01190) expression according to the Simon equation (Simon, 2003). Bars represent means ± SE of three biological replicates. All genes in all genotypes resulted significantly modulated (*t*-test; *p* < 0.05) between C and PFD berries. The † indicates no significance.

The expression of the seven selected genes was investigated by qPCR in C and PFD berries of Nero d’Avola, Ortrugo, and Ciliegiolo during 2012, only at the corresponding stage. We found that flavonol synthase at Stage 1, jasmonate *O*-methyltransferase at Stages 2 and 3 and the ABA receptor PYL4 at Stage 4 confirmed the modulation of expression obtained by microarray analysis in Sangiovese in BO and AN (**Figure 5**). Instead, the MADS-box AGL20, ABC transporter and VvTPS62 were not commonly modulated by PFD in all genotypes (data not shown). The expression of flavonol synthase, jasmonate *O*-methyltransferase and the ABA receptor PYL4 was then evaluated by qPCR in C and PFD berries of all genotypes during 2013. They all showed the same modulation after treatment in all genotypes and growing conditions (**Figure 6**), resulting as good candidates for molecular markers of the PDF treatment.

Concerning the common upregulation of flavonol synthase, we determined main berry flavonols at harvest in both 2012 and 2013 on cvs Ortrugo and Sangiovese, evidencing a clear pattern of a significant increase in PFD treatment in the two sites and genotypes (**Table 5**). This trend was overwhelming vs. year-to-year variability and especially marked for quercetin 3-*O* glucuronide + quercetin 3-*O* glucoside and kaempferol 3-*O* glucoside.

**Table 5:** Main flavonols concentration.

	Quercetin 3-*O*-glucoronide (mg/kg)		Quercetin 3-*O*-glucoside (mg/kg)		Myricetin (mg/kg)		Kaempferol 3-*O*-glucoside (mg/kg)	
Cultivar and Year	Control	PFD	Control	PFD	Control	PFD	Control	PFD
Ortrugo 2012^1^	314b	447a	202b	327a	16.6	23.3	35.1	45.3
Ortrugo 2013^1^	475b	759a	199b	402a	17.0b	39.7a	37.1b	68.2a

	**Quercetin 3-*O*-glucoronide + Quercetin 3-*O*-glucoside (mg/kg)**				**Myricetin (mg/kg)**		**Kaempferol 3-*O*-glucoside (mg/kg)**	
**Cultivar and Year**	**Control**		**PDF**		**Control**	**PFD**	**Control**	**PFD**
Sangiovese 2012 ^2^	260b		567a		94.4	129.4	11.6b	41.1a
Sangiovese 2013 ^2^	263b		523a		63.2	94	16b	40a

*Concentration of quercetin, myricetin, and kaempferol recorded at harvest in berries of cvs Ortrugo and Sangiovese (Bologna site) vines subjected to PFD or undefoliated (control) in 2012 and 2013. Data are given as mean ± standard error (SE). Mean separation within row and parameter by t-test, P < 0.05. Absence of letters means ns. ^1^Data are given on a skin dry weight basis. ^2^Data are given on a berry fresh weight basis.*

These data strongly support the involvement of flavonol synthase in the PFD response in berry independently of growing site, year, and genotype.

## Discussion

### Influence of Growing Site, Year, and Genotype on the Defoliation Response

The testing of four different genotypes over two consecutive years and having also compared a cultivar (Sangiovese) in the same year under two growing conditions (BO and AN) permits a proper discussion about the interactive effects between the above factors on vine response to PFD applied in all instances with the same protocol (i.e., six main basal leaves removed at the “separated closed flowers” stage with retention of any laterals). Growing conditions (BO vs. AN) exerted an overall moderate effect on vine response variability to early leaf removal. It is notable that, in 2012, although AN vines were clearly under-cropped as compared to BO, variations of significantly modified parameters were always the same, while the same also held true for unmodified parameters, with the exception of TA and total anthocyanins (**Table 1**). In 2013, the influence of growing conditions was greater, albeit essentially limited to berry weight that was greatly reduced at BO site, while it was unchanged at AN. Since the vine balance given as LA/yield did not change in the two locations, it is likely that the improved total anthocyanins concentration at harvest in BO grapes results from inherently smaller berry size, hence higher skin-to-pulp ratio. This hypothesis is well supported by results obtained in a previous study showing that berries from pre-flowering defoliated Sangiovese vines, characterized by a significant increase of anthocyanin concentration in comparison to the control, also demonstrated a significant increase of berry skin thickness (Pastore et al., 2013).

Variability in the response to PFD attributable to year was, in AN and BO sites, more pertinent to must composition than to vegetative growth and yield parameters. In particular, TSS was more responsive in 2013, showing a large increase in PFD vines, whereas TSS at both sites was unchanged in 2012. The reasons for this difference are not easy to distinguish; however, the quite high TSS reached in AN in 2012 likely reflects the high LA/yield (>1.9 m^2^/kg) placing no limitations on the sugar accumulation process; in confirmation of this, PFD was more effective in increasing TSS in 2013 at quite low LA/yield ratio, suggesting that, in both sites, PFD might have benefitted from higher foliage “quality” due to lower canopy age from veraison onward (Poni et al., 2006). In the other sites, response to PFD over the 2 years was more consistent for all the parameters considered.

Comparing four genotypes over 2 years yielded a total of 10 C vs. PFD comparisons. All genotypes showed high responsiveness to the technique and no “recalcitrant” varieties could be discriminated. In more detail, in all cases yield per vine was significantly reduced after the early defoliation; among the main yield components cluster weight was reduced in 9 out of 10, berry weight in 5 out of 10, and berries per cluster in 6 out of 10. This confirms previous studies that PFD is extremely effective and consistent at reducing yield through either lower fruit set or smaller berry size or a combination of both, in turn resulting in looser clusters. This is not a surprising finding since the physiological background on which the technique relies is quite robust; it is well-known from the literature (Coombe, 1962; Hardie and Considine, 1976) that a calibrated source limitation imposed pre-flowering constrains the carbohydrate pool available to support flowering and fruit-set which, inherently, become limited. In terms of grape composition, it is likewise confirmed that, with very few exceptions, all genotypes subjected to PFD show a significant increase in TSS at harvest (7 cases out of 10) and, for red cultivars, in total anthocyanins (6 out of 8). The physiological bases for such response are also quite solid: (i) in PFD, ripening benefits from non-limiting or even higher final LA/yield ratios since induced yield limitation is often greater than the amount of leaf area removed “*per se*” with defoliation; (ii) as reported in Poni et al. (2006), in PDF the amount of carbohydrate supply per unit of grape fresh mass is higher from veraison onward due to an overall younger, hence more efficient canopy, and compensation mechanisms in either leaf area development or maximum photosynthetic rates and (iii) TSS and total anthocyanins can also be enhanced due to smaller berry size. Another quite remarkable and consistent feature of the PDF practice was that, despite the large increase in TSS, TA was reduced only in the Ortrugo trial, in five out of eight cases it was unchanged and on one occasion (AN, 2012) it was increased. This suggests that the technique is quite effective at decoupling the sugar/acid ratio and if TSS are increased, TA is not necessarily concurrently decreased. This feature is of special interest within a global warming scenario (Palliotti et al., 2014), where maintenance of adequate acidity in warm environments is an increasing concern.

### Common Molecular Responses to Pre-flowering Defoliation in Sangiovese Berries Involved Genes Related to Secondary and Hormone Metabolism

Berry molecular responses to PFD were initially investigated by global gene expression analysis performed on berries at four developmental stages in defoliated and control Sangiovese vines, grown in BO and AN in 2012. Statistical analysis clearly revealed that the environment has a stronger effect than defoliation treatment on the transcriptome rearrangement during berry development in Sangiovese. Indeed, the correlation dendrogram showed a very clear distinction between BO and AN berry transcriptomes at each developmental stage and a weak and variable separation between berries from PFD and C vines. In addition, a PCA analysis revealed that no principal components were able to distinguish, at transcriptional level, berries from PFD and C vines. PCA analysis also showed that the ripening process was slightly advanced in AN in comparison to BO, reflecting the higher level of TSS, lower values of TA and higher anthocyanin content observed in ripe berries grown in AN, independently of the PFD effect (**Table 1**).

It is very unlikely that inter-clonal variation within the same genotype might have interfered in the transcriptional responses. Albeit some slight differences in berry transcriptome during development can be attributable to small variations in the sampling procedure adopted in the two locations, the growing site seems to strongly affect berry gene expression during development in Sangiovese, evidencing that a large part of its transcriptional ripening program is plastic. Previous attempts to quantify the transcriptomic plasticity in grapevine berry were recently reported for the red cultivar Corvina and white cultivar Garganega (Dal Santo et al., 2013a, 2016b). These studies demonstrated a wide berry phenotypic plasticity in both cultivars, in particular affecting the secondary metabolism, suggesting that this phenomenon, which allows the production of different wines from the same cultivar and the adaptation of the same cultivar to diverse growing regions, is still scarcely characterized in grapevine.

Sangiovese is the top red variety grown in Italy with about 70.000 hectares (ISTAT, 2015), and is cultivated in several regions (i.e., Tuscany, Emilia Romagna, Marche, Umbria). It is well known that Sangiovese wine made with grapes from Emilia Romagna usually reaches a price tag that is 5–10 times lower than any Brunello di Montalcino label, which is likewise made with 100% Sangiovese grapes. These differences might reflect consumer perception and expectations as well as marketing strategies. In this context our transcriptomic survey, reflecting the growing site effect of two distinct Italian regions (Emilia Romagna and Marche), should be further explored to unveil the genotype × environment interactions of this important Italian grapevine cultivar.

The impact of the environment on Sangiovese berry transcriptome was further highlighted by the analysis of DEGs in berries from C and PFD vines in the two sites. Among the 1746 and 1041 DEGs found in BO and AN, respectively, only 125 were commonly differentially expressed, strongly suggesting that the effect of PFD on berry gene expression is mainly affected by growing conditions.

The GO enrichment analysis performed on these commonly DEGs revealed that “response to stimulus” and “cellular amino acid and derivative metabolic process” functional categories were significantly over-represented. The same two categories were previously found overrepresented in the list of genes differentially expressed at the end of veraison in Sangiovese berries subjected to both pre-flowering and veraison defoliation treatment (Pastore et al., 2013). The role in stress resistance of many genes involved in amino acid metabolism, such as the aspartate aminotransferase, glutamine synthetase, and serine hydroxymethyltransferase, was previously described (Moreno et al., 2005; Singh and Ghosh, 2013; de la Torre et al., 2014; Wu et al., 2016), strongly suggesting that stress response induction is one of the principal effects of PFD on berry transcriptome, independently of the environment. Interestingly, other functional categories were found among the 125 genes, predominantly the “secondary metabolic process,” mainly represented by genes belonging to the phenylpropanoid pathway, and “hormone stimulus,” with several genes related to auxin, ethylene, and ABA metabolism. These results support previous observations regarding the direct effect on the expression of genes involved in berry ripening exerted by leaf removal (Pastore et al., 2013). Moreover, the modulation of phenylpropanoid-related genes in defoliated berries could represent a stress response, as previously observed in grapevine upon various stresses (Matus et al., 2009; Rienth et al., 2014; Corso et al., 2015).

In order to identify putative berry molecular markers of the PFD treatment, we focused only on genes characterized by a low plasticity during berry development. Among these, six were selected as putative markers of the treatment, being similarly modulated throughout berry development by PFD in the two sites and for their possible role in berry formation and ripening.

The expression profiles of these candidates were analyzed by qPCR in berries from PFD and C vines in BO and AN in 2012, in order to validate microarray data, and in 2013 in order to assess the vintage effect on their expression modulation after treatment. Five out of six selected genes demonstrated a consistent modulation of expression induced by PFD throughout berry development in both years and in both sites, emerging as putative molecular markers for this treatment in the cultivar Sangiovese.

The ABC transporter VvPDR20/VvABCG50 showed a delay in its peak of expression at Stage 3, when berries are softening and start to accumulate pigments, instead of Stage 2, when berries are still green and firm. ABC transporters superfamily constitutes one of the largest families of transmembrane proteins that plays important roles in the vacuolar accumulation of secondary metabolites, such as flavonoids, in detoxification and heavy metal sequestration, in chlorophyll catabolite transport and ion channel regulation (Klein et al., 2006; Kang et al., 2010). In grapevine, 135 putative ABC proteins were identified and classified (Cakir and Kilickaya, 2013). The VvPDR20/VvABCG50 gene belongs to the PDR subfamily in *V. vinifera*, the largest ABC transporter subfamily. In grapevine, no PDR-related ORF has been cloned in its entirety and characterized (Cakir and Kilickaya, 2013). However, in other species, members of this family confer resistance to various biotic and abiotic stresses (Moons, 2003; Lee et al., 2005; Stukkens et al., 2005). Interestingly, it was recently shown in Arabidopsis that PDR12 is a plasma membrane ABA uptake transporter that mediates cellular uptake of the phytohormone ABA in guard cells (Kang et al., 2010). VvPDR20/VvABCG50 gene is an interesting molecular marker directly affected by the PFD treatment in Sangiovese, and is a good candidate for future functional studies.

The indole-3-acetate beta-glucosyltransferase and the auxin responsive factor 10, involved in auxin metabolism and signaling, respectively, were also identified as PFD molecular biomarkers. It is generally acknowledged that auxin plays a role in fruit growth. However, the change in auxin levels during berry development and the dynamics of auxin transport and signaling are still under debate (Fortes et al., 2015). It has been demonstrated that auxin treatment of pre-veraison grape berries delays ripening and alters the expression of developmentally regulated genes, suggesting that low auxin levels are required to trigger the onset of ripening (Davies et al., 1997; Bottcher et al., 2011; Ziliotto et al., 2012). However, it has been also hypothesized that high auxin levels at pre-veraison stages are required for the induction of genes involved in the ripening inception (Ziliotto et al., 2012; Corso et al., 2016). The regulation of auxin levels is associated with the conjugation of indole acetic acid (IAA) by the indol-3-acetate beta-glucosyltransferase, which allows an increase in the conjugated form of auxin after veraison. The decrease of expression after veraison of the indol-3-acetate beta-glucosyltransferase gene, obtained in C Sangiovese vines in the 2 years, corroborates previous results obtained in three Portuguese varieties (Agudelo-Romero et al., 2013) and supports the role of this gene in auxin level regulation during ripening. Interestingly, an increase in expression level of this gene was found as a consequence of PFD treatment, which delays the gene downregulation, possibly affecting the IAA homeostasis and, hence, the regulation of the onset of ripening.

Auxin response factors regulate the auxin-mediated gene expression (Tiwari et al., 2003). In grapevine, 19 ARF genes were recently identified (Wan et al., 2014; Corso et al., 2016) but no functional studies have been performed to date. The ARF 10 exhibited a peak of expression at the ripening stage in C vines, whereas the peak of expression was hastened at the end of veraison in PFD vines (Stage 3), strongly suggesting that the treatment can interfere with the ripening progress by affecting the auxin level at the end of veraison. Biochemical analyses aimed to quantify the free and conjugate auxin form in berries from PFD and C vines will be necessary to thoroughly characterize the possible impact on hormonal regulation exerted by the PFD.

The last two putative PFD molecular markers identified in Sangiovese were a CAD gene, involved in the last step of the synthesis of the monomeric precursors of lignin, and a G3T, involved in the glycosylation of flavonols. Both genes showed an increase of expression in berries from PFD vines, more pronounced at Stage 2 in Bologna and at Stage 3 in Ancona. The increase in expression of CAD was already observed in Sangiovese berries from PFD vines (Pastore et al., 2013). It was previously proposed that the increase in sunlight exposure of berries on defoliated vines induced the expression of genes involved in cell wall metabolism that allow an increase in berry skin thickness, providing more epidermal layers for protection against sunburn and storage of anthocyanin compounds (Pastore et al., 2013).

The G3T coincides with the previously characterized VvGT6, contributing to flavonol glycosylation (Ono et al., 2010). The upregulation of this gene obtained in berries from PFD vines is consistent with the higher flavonol content that berries subjected to PFD manifested (Pastore et al., 2013; this work).

Among the six genes, only the expression of geraniol 10-hydroxylase, a cytochrome P450 monooxygenase involved in the biosynthesis of terpenoids (Collu et al., 2001) and phenylpropanoids (Sung et al., 2011), showed an inconsistent modulation of expression between the 2 years. Differences in expression trend of this gene and its responsiveness to the defoliation treatment could be linked to the differences in weather patterns between 2012, marked by a long summer with hot spells, and the slightly cooler 2013. It is well known that terpenoids metabolism contributes to plant adaptation to the environment, in particular solar exposure, UV-B radiation (Gil et al., 2013; Zhang et al., 2014) and drought (Deluc et al., 2009; Selmar and Kleinwachter, 2013; Savoi et al., 2016).

### General Molecular Markers of the Pre-flowering Defoliation

Expression analysis of the PFD Sangiovese putative markers was performed on another three genotypes subjected to the same treatment in different environments in 2012. A stronger influence of genotype and/or growing site than PFD treatment on these genes’ expression was clearly revealed during berry development, indicating that they should not be considered as PFD markers for genotypes other than Sangiovese. We therefore selected new candidates by focusing on DEGs at each berry developmental stage, without evaluating the entire expression profile.

The inclusion in our experimental plan of an early stage of berry development (Stage 1), allowed previous transcriptomic results, focused only on the PFD effect on berry ripening phase, to be greatly improved (Pastore et al., 2013). Indeed, several DEGs in PFD berries in comparison to C were found at Stage 1 in both sites. This strongly supports recent results obtained in defoliated Cabernet Sauvignon berries showing that earlier berry stages react to leaf removal distinctly from the later developmental stages (Young et al., 2016).

A flavonol synthase resulted as one of the most upregulated genes in PFD Sangiovese berries at Stage 1 in both sites and its upregulation was confirmed by qPCR in all genotypes, environments, and years. Interestingly, the upregulation of the same gene was previously observed in both pre-flowering and veraison defoliated Sangiovese berries at the end of veraison (Pastore et al., 2013) and the expression of two isoforms of flavonol synthase was affected in defoliated berries of Cabernet Sauvignon (Matus et al., 2009). Quantification of the main berry flavonols at harvest in Ortrugo and Sangiovese in both years evidenced a significant increase in berries from PFD vines, in the two sites, genotypes and years, strongly supporting transcriptomic data. In Sangiovese PFD treated berries, the concentration of quercetin, the main flavonol in red grapes (Mattivi et al., 2006), and kaempferol, was more than twice that in control, in both years, whereas the increase of myricetin at harvest was less intense, as previously observed (Pastore et al., 2013). This shift in flavonol composition was not observed in Ortrugo, in which the abundance of all flavonol compounds increased after PFD treatment in both years, as previously reported for Merlot (Spayd et al., 2002). It was demonstrated that the induction of flavonols synthesis is positively correlated to sunlight exposure, reflecting their role as UV protectants (Price et al., 1995; Haselgrove et al., 2000; Downey and Rochfort, 2008; Matus et al., 2009). Our data suggested that the PFD-induced expression of flavonol synthase gene at an early stage of berry development is due to an increase in cluster sunlight exposure, causing a significant accumulation of flavonols in berries at harvest, and that this effect is shared across different environments, years, and genotypes.

A jasmonate *O*-methyltransferase gene resulted as being another common molecular marker of the defoliation for both Stage 2 and Stage 3 of berry development, being positively modulated by PFD in all tested conditions. The role of methyl jasmonate (MeJA) in the response to biotic and abiotic stresses was widely discussed in the past (Cheong and Choi, 2003; Wasternack and Song, 2016). In non-climacteric fruits such as strawberry and grape, JA levels are reported to be high in early development and decreasing to lower values in riper fruits, enabling the onset of ripening to occur (Kondo and Fukuda, 2001). In grapevine, the gene coding for jasmonate *O*-methyltransferase was found downregulated in ripe fruits of three grape varieties (Agudelo-Romero et al., 2013). Interestingly, the downregulation of jasmonate *O*-methyltransferase during berry development was revealed by transcriptomic analysis on Sangiovese berries from C vines in both Bologna and Ancona in 2012. As a consequence, the upregulation observed at Stages 2 and 3 in berries from PFD vines corresponded to a delay in its downregulation and not to a genuine induction.

It was recently demonstrated that JA plays an important role in grape berry coloring and softening by inducing the transcription of several ripening-related genes, such as phenylpropanoid genes, cell wall metabolism-related genes and genes involved in aroma accumulation (Jia et al., 2016). Consistently, we observed that at Stage 2 and Stage 3 several terpenoid synthase genes (e.g., one geraniol 10-hydroxylase and several different terpene synthases), involved in berry aromatic compounds accumulation, was found commonly upregulated by PFD in Bologna and Ancona in 2012, together with jasmonate *O*-methyltransferase. Intriguingly, at Stage 3 also a jasmonate ZIM-Domain VvJAZ2 gene, involved in JA signaling cascade (Pauwels and Goossens, 2011; Wasternack and Song, 2016), resulted as commonly modulated by PFD in Sangiovese berries in both sites. Although the expression of this gene was not assessed in all conditions, we could hypothesize that the PFD impacts on berry ripening, possibly affecting the JA metabolism at veraison, when the JA level has recently been proposed to play a crucial role in ripening regulation (Jia et al., 2016).

The last identified berry PFD marker was the ABA receptor PYL4, which resulted as being the most commonly downregulated gene at Stage 4 in Sangiovese in 2012. Interestingly, all genes commonly modulated in PFD Sangiovese berries at Stage 4 in that year resulted as downregulated, suggesting that the treatment could affect many metabolisms by hastening their normal shutdown. This hypothesis also holds true for the ABA receptor PYL4, showing a decreasing expression trend during ripening in C conditions with a peak of expression at veraison, and an accelerated downregulation in PFD berries. The ABA receptor PYL4 belongs to the PYR/PYL/RCAR receptors family that, together with the PP2Cs and SnRK2s kinases, constitutes the complex molecular machinery involved in the ABA-mediated signaling pathway (Boneh et al., 2012).

Many studies indicated that ABA, together with other phytohormones like brassinosteroids (BRs) and ethylene may play an important role in several ripening-associated processes of grape berry (Davies and Bottcher, 2009; Kuhn et al., 2014). It was observed that free ABA levels increase around veraison, concurrently with sugar accumulation, berry coloration, and softening, whereas during ripening ABA levels may be controlled mainly by conjugation to glucose. Nevertheless, it was found that a set of genes involved in the ABA-mediated signaling pathway, including the ABA receptors PYL8 RCAR3 and PYL9 RCAR1, were upregulated at the mid-ripening phase in three Portuguese varieties (Fortes et al., 2011; Agudelo-Romero et al., 2013). This expression trend was consistent with the expression of PYL4 found in C vines, suggesting that later in ripening, ABA synthesis is not induced, but ABA-regulated processes are instead activated. The lower expression of PYL4 observed in PFD berries at harvest, which corresponds to a faster downregulation in comparison to C, suggests that PFD treatment hastened the ABA-mediated ripening signaling possibly by inducing abiotic stress response early during development. This faster downregulation of PYL4 is particularly marked in Ortrugo cultivar, in which the highly significant increase in ripening parameters in both years suggests that the ripening process in this cultivar started much earlier than in the other cultivars in the berries after treatment.

## Conclusion

A comparison was made of physiological and molecular responses to PFD in four grapevine cultivars grown in different Italian geographical areas and during two consecutive years to evaluate the interactive effects between these factors on vine response to PFD and to determine the common effect of defoliation in berry at transcriptional level. All genotypes were highly responsive to the technique, the yield per vine being significantly reduced in all conditions. In terms of grape composition, a significant increase in sugar content and total anthocyanins at harvest was obtained in most genotypes. Sangiovese resulted as being the cultivar with the stronger variability in must composition in the response to defoliation. Global gene expression analysis performed on Sangiovese berries from defoliated and untreated control vines grown in two different sites highlighted, on the one hand, the strong effect of environment on the berry transcriptional ripening program in this cultivar and, on the other, allowed genes commonly regulated by selective leaf removal to be identified. The differential expression of these putative marker genes, mainly related to secondary metabolism and hormone signaling, could link the defoliation treatment to physiological and metabolic changes found in treated berries. These new insights greatly improve previous knowledge about molecular mechanisms on the basis of the qualitative outcomes of an important and widely used management technique in viticulture, allowing physiological responses in berry to the selective PFD practice to be precisely defined across genetic and environmental variability.

## Author Contributions

SZ designed the microarray experiments, interpreted the micro array data, and wrote the manuscript, SDS performed the statistical analyses, SDS and GBT interpreted the microarray data and helped in drafting the manuscript, ED performed the RNA extraction, microarray experiments, and real-time qPCR analysis, IF, CP, GA, OS, VL, APi, RDL, APa, ST and MG conducted defoliation experiments, sampled the material and processed the data, CP, GA and MG performed the HPLC analysis, SP conceived and supervised the study, wrote and critically revised the manuscript. All authors contributed to the revision of the manuscript.

## Conflict of Interest Statement

The authors declare that the research was conducted in the absence of any commercial or financial relationships that could be construed as a potential conflict of interest.

## Footnotes

1 http://bioinfo.cau.edu.cn/agriGO

2 https://www.ncbi.nlm.nih.gov/geo/query/acc.cgi?acc=GSE92980
