# Supplementary material for: Transcriptional Responses to Pre-flowering Leaf Defoliation in Grapevine Berry from Different Growing Sites, Years, and Genotypes
Source: Front Plant Sci. 2017 May 2;8:630. doi: 10.3389/fpls.2017.00630 (PMC5411443; doi:10.3389/fpls.2017.00630)
Supplement: Supplementary file 1 [file Table_1.PDF]

Supplementary Table 1. List of primer sequences used in qPCR analyses

Gene ID	Primer name	Sequence 5'- 3'
VIT_06s0061g01490	ABC transporter VvPDR20-VvABCG50 FOR<br>ABC transporter VvPDR20-VvABCG50 REV	AGGTCGACACTCTCACAAAGCTTGTTG<br>AGCCTCAACTGCCGAACTGCTGATC
VIT_13s0019g04380	Auxin response factor 10 FOR<br>Auxin response factor 10 REV	ATTCGGCAGCAACACCCTA<br>CTCCCATCACTCCCAAAAGC
VIT_00s0615g00020	Cinnamyl alcohol dehydrogenase FOR<br>Cinnamyl alcohol dehydrogenase REV	GTTCCAATGGACTATGTAAACACTGCATTG<br>TTGTCCTAGGCAGCTTTCAGCGTG
VIT_11s0052g01630	Flavonoid 3-O-glucosyltransferase FOR<br>Flavonoid 3-O-glucosyltransferase REV	AGGGAACATGCAATGGACAA<br>TCCAACTGAGGCATGTGCTA
VIT_15s0048g01490	Geraniol 10-hydroxylase FOR<br>Geraniol 10-hydroxylase REV	AGTGGCCAGCAAGTGTGCTA<br>AGGGTCGGGAGTCAGATAAGG
VIT_13s0019g03040	Indole-3-acetate beta-glucosyltransferase FOR<br>Indole-3-acetate beta-glucosyltransferase REV	AAGATTGTTGAGCGCCAGAT<br>ACGCCATCTGAAAAGGTCAG
VIT_18s0001g03470	Flavonol synthase FOR<br>Flavonol synthase REV	ATGCTGCTTGGTGTGTGAGA<br>TCGCATAGGATCGGGTACTT
VIT_18s0001g12890	Jasmonate O-methyltransferase FOR<br>Jasmonate O-methyltransferase REV	CTGTGGTTGAGTCCATGCTA<br>CCCTTGTCTTTGCCAAGTGA
VIT_08s0058g00470	Abscisic acid receptor PYL4 RCAR10 FOR<br>Abscisic acid receptor PYL4 RCAR10 REV	GATCCGATGCGCTCCATAC<br>CGTCGGAAAGCGATAAACAAA
VIT_16s0098g01190	Ubiquitin FOR<br>Ubiquitin REV	TCTGAGGCTTCGTGGTGGTA<br>AGGCGTGCATAACATTTGCG
