# Supplementary material for: Transcriptional Responses to Pre-flowering Leaf Defoliation in Grapevine Berry from Different Growing Sites, Years, and Genotypes
Source: Front Plant Sci. 2017 May 2;8:630. doi: 10.3389/fpls.2017.00630 (PMC5411443; doi:10.3389/fpls.2017.00630)
Supplement: Supplementary file 7 [file Image_1.PDF]

Supplementary Figure 1.

A

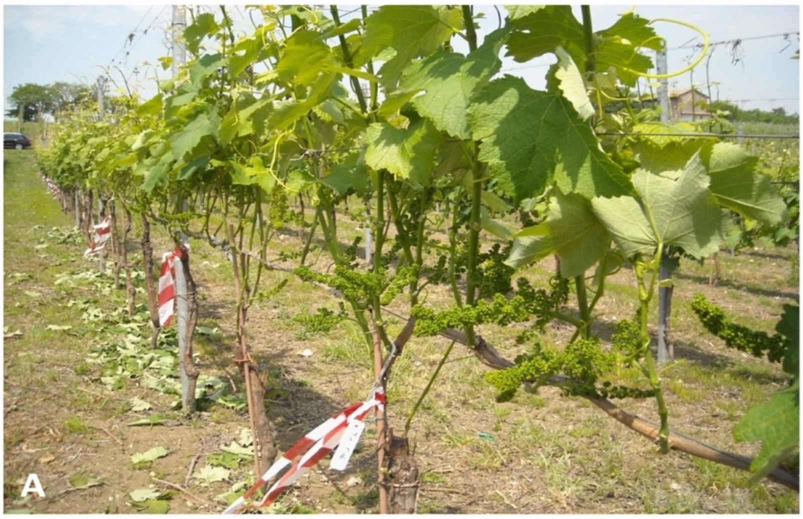

B

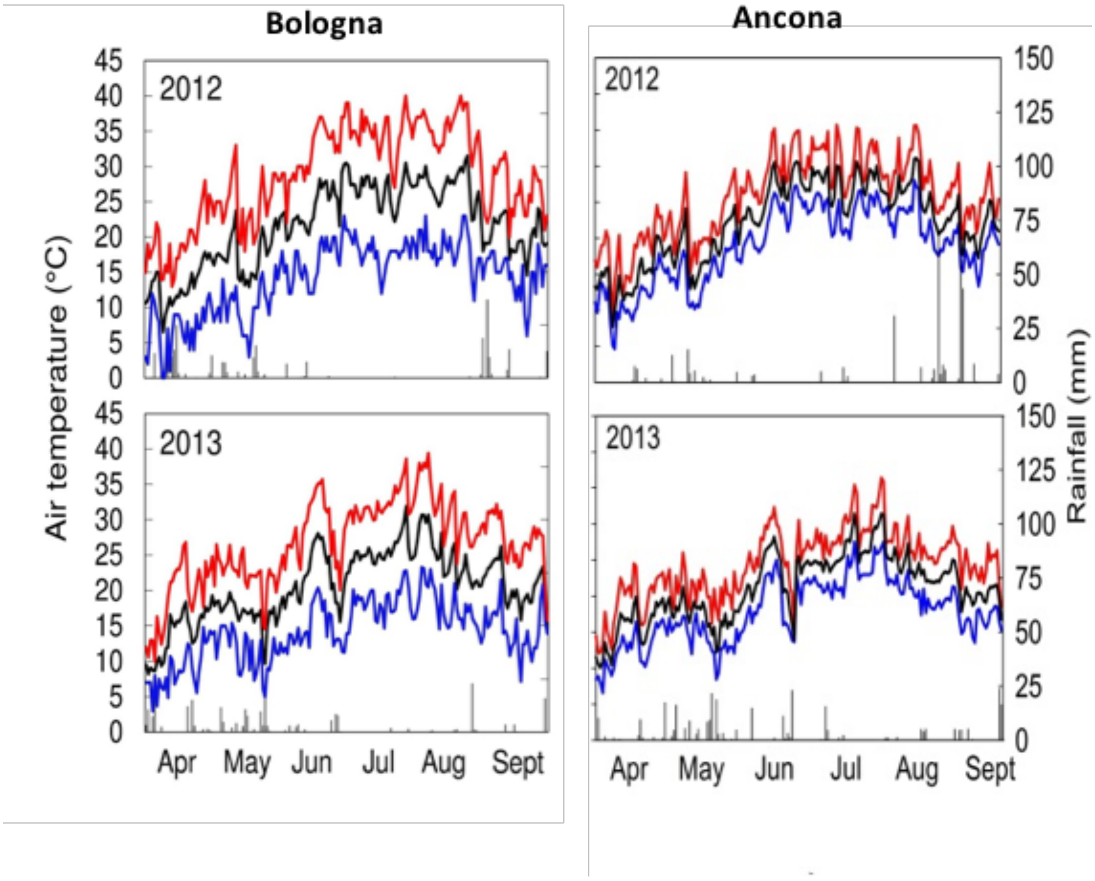

Supplementary Figure 1. A. Image of PFD vines. B. Daily mean (black), minimum (blue) and maximum (red) air temperature (T) recorded in 2012 and 2013 at Bologna and Ancona locations from 1 April till 30 September. Vertical bars indicate daily rainfall.
