# Supplementary material for: Transcriptional Responses to Pre-flowering Leaf Defoliation in Grapevine Berry from Different Growing Sites, Years, and Genotypes
Source: Front Plant Sci. 2017 May 2;8:630. doi: 10.3389/fpls.2017.00630 (PMC5411443; doi:10.3389/fpls.2017.00630)
Supplement: Supplementary file 8 [file Image_2.PDF]

Supplementary Figure 2.

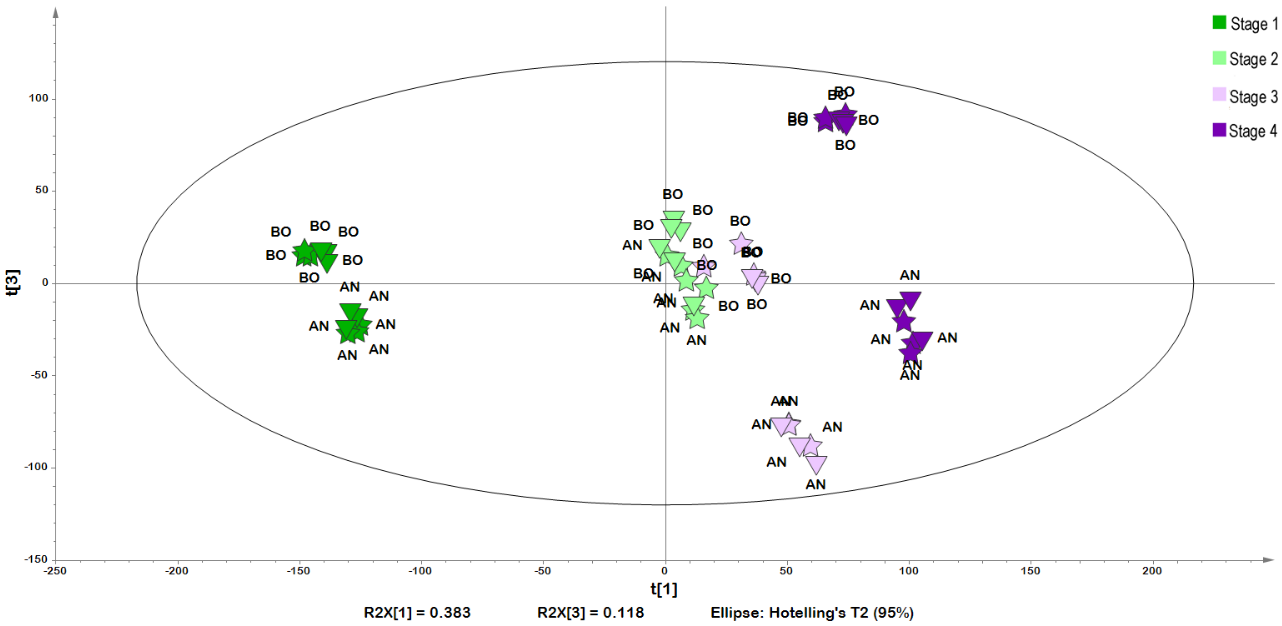

Supplementary Figure 2. Principal component analysis of the differentially expressed genes dataset (PC1 vs. PC3). The score scatter plot of the PCA model (9 components, R2X(cumulative) = 0.903, Q2 (cumulative) = 0.848) was generated using Simca P+ 13.0 and coloured according to the stage of development. Different treatments are indicated by different symbols, "☆"=Control "▽"=Pre-flowering defoliation"
